# Targeting the NLRP3 inflammasome–IL-1β pathway in type 2 diabetes and obesity

**DOI:** 10.1007/s00125-024-06306-1

**Published:** 2024-11-04

**Authors:** Daniel T. Meier, Joyce de Paula Souza, Marc Y. Donath

**Affiliations:** 1 Clinic of Endocrinology, Diabetes and Metabolism, University Hospital Basel, Basel, Switzerland; 2 Department of Biomedicine, University of Basel, Basel, Switzerland

**Keywords:** Clinical study, Diabetes, IL-1β, Inflammasome, Inflammation, Insulin, NLRP3, Obesity, Pancreatic islet, Review

## Abstract

Increased activity of the NACHT, LRR and PYD domains-containing protein 3 (NLRP3) inflammasome–IL-1β pathway is observed in obesity and contributes to the development of type 2 diabetes and its complications. In this review, we describe the pathological activation of IL-1β by metabolic stress, ageing and the microbiome and present data on the role of IL-1β in metabolism. We explore the physiological role of the IL-1β pathway in insulin secretion and the relationship between circulating levels of IL-1β and the development of diabetes and associated diseases. We highlight the paradoxical nature of IL-1β as both a friend and a foe in glucose regulation and provide details on clinical translation, including the glucose-lowering effects of IL-1 antagonism and its impact on disease modification. We also discuss the potential role of IL-1β in obesity, Alzheimer’s disease, fatigue, gonadal dysfunction and related disorders such as rheumatoid arthritis and gout. Finally, we address the safety of NLRP3 inhibition and IL-1 antagonists and the prospect of using this therapeutic approach for the treatment of type 2 diabetes and its comorbidities.

**Graphical Abstract:**

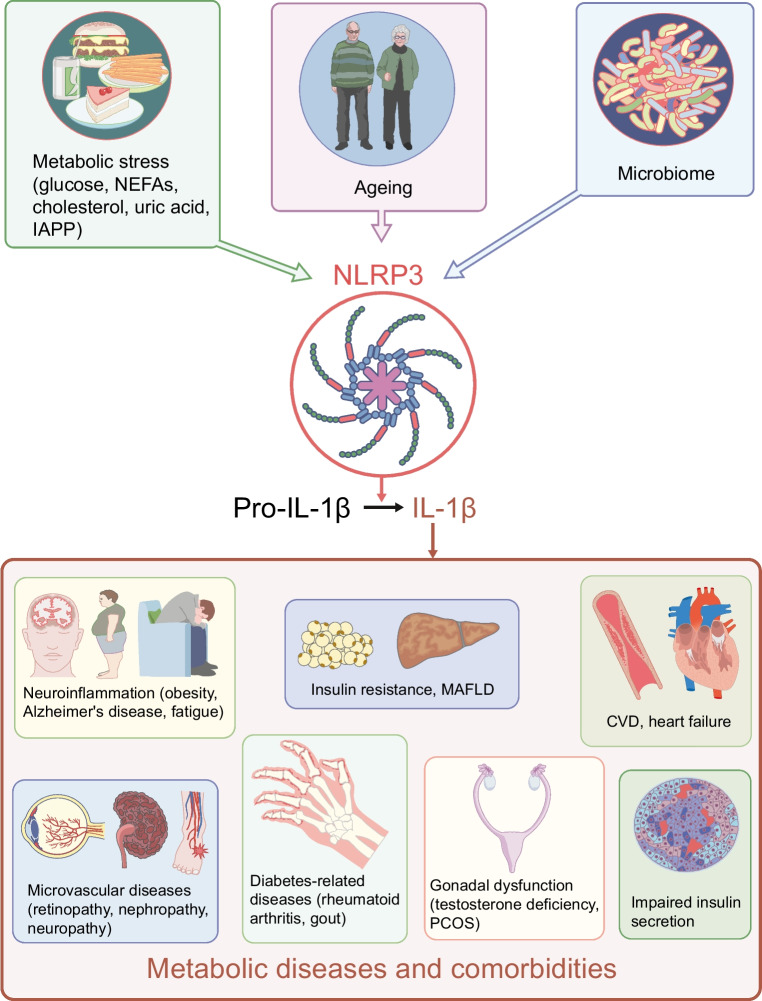

**Supplementary Information:**

The online version contains a slideset of the figures for download available at 10.1007/s00125-024-06306-1.

## Historical perspective

The discovery of a ‘fever-inducing factor’, later renamed IL-1β, dates back to 1943 when Eli Menkin showed that supernatants of leucocytes isolated from pus were pyrogenic when injected into rabbits. Using a similar approach, Paul Beeson confirmed that endotoxin-free proteins induce fever [1]. In 1977, Charles Dinarello and colleagues identified a highly potent human leucocytic pyrogen [2], which, together with Philip Auron and colleagues, allowed the cloning of human IL-1β in 1984 [3], leading to the proof that recombinant IL-1β induces fever in humans [4]. In the same year, the related IL-1α was cloned [5] and, in 1990, the naturally occurring IL-1 receptor antagonist (IL-1Ra) followed [6], leading to the conclusion that there are three proteins that compete for binding to the IL-1 receptor [7].

In the following years, seminal discoveries relating to IL-1β maturation were published. As such, IL-1β-converting enzyme (later renamed caspase 1) was discovered and shown to be required to proteolytically cleave pro-IL-1β to yield a biologically active protein [8]. A decade later, cryopyrin (NACHT, LRR and PYD domains-containing protein 3, also known as NLRP3) was identified in individuals with autoinflammatory Muckle–Wells syndrome [9], and the adaptor protein apoptosis-associated speck-like protein containing a caspase recruitment domain (ASC, also known as PYCARD) was implicated in apoptotic processes [10]. This paved the way for the discovery of the inflammasome in the laboratory of Jürg Tschopp [11] in 2002.

## Molecular mechanism of the activation and assembly of the NLRP3 inflammasome and activation of IL-1β and IL-18

**Fig. 1:**
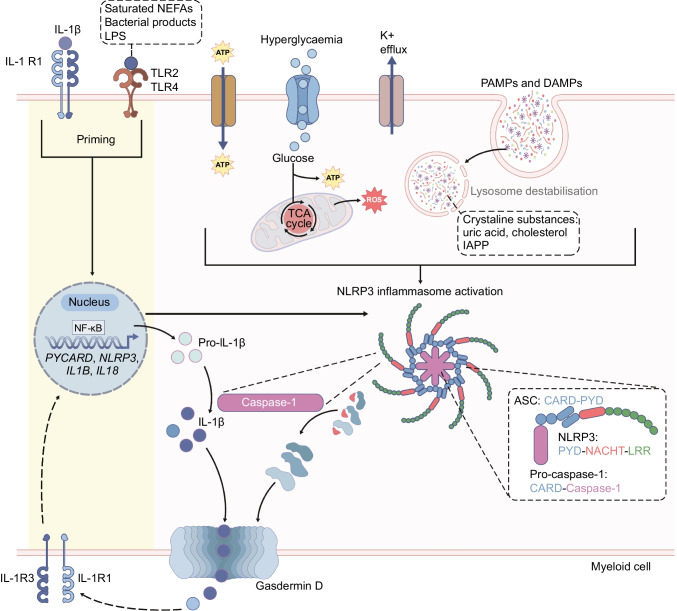
Schematic overview of canonical IL-1β production and secretion. TLR2, TLR4 and IL-1 signalling prime myeloid cells, starting the transcription of inflammasome components (e.g. *PYCARD* [ASC], *NLRP3*) and IL-1-responsive genes (e.g. *IL1B*, *IL1RN* [IL-1RA], *CXCL8* [IL-8], *IL6*). Glucose, K^+^ efflux, and DAMPs and PAMPs trigger intracellular stimuli that act as a second signal activating the NLRP3 inflammasome. Elevated cytosolic levels of reactive oxygen species (ROS), islet amyloid polypeptide (IAPP) and ATP activate NLRP3 receptors. ROS and crystalline substances such as cholesterol and uric acid disrupt the lysosomal membrane in phagocytes, promoting leakage of lysosome cargo into the cytosol, releasing epitopes that are recognised by the LRR domain of the NLRP3 receptor. The NLRP3 inflammasome is composed of the sensor NLRP3, the adaptor protein ASC and the effector molecule caspase-1. On receiving a second stimulus, the NLRP3 inflammasome is assembled in the cytosol, leading to the activation of caspase-1 and subsequent secretion of active IL-1β via the gasdermin D pore. After being secreted, IL-1β binds to the IL-1R1, amplifying intracellular inflammatory signalling and transcription of NLRP3 inflammasome components. TCA, tricarboxylic acid. This figure is available as part of a downloadable slideset

The seminal discovery of the ‘inflammasome’ protein complex in 2002 started a new era of study into the pathological mechanisms associated with the innate immune system and inflammation. Currently, several inflammasome complexes have been identified, with the NLRP3 inflammasome, which is highly expressed in myeloid cells, viewed as the most relevant for detecting metabolic changes. NLRP3 inflammasome activation requires two steps—priming and assembly—although some caspase-1-dependent IL-1β can be released in the absence of the latter step [12]. In the priming step, pathogen-associated molecular patterns (PAMPs, e.g. lipopolysaccharide [LPS]), damage-associated molecular patterns (DAMPs, e.g. ATP, reactive oxygen species, K^+^ efflux, crystalline substances such as cholesterol, uric acid and human islet amyloid polypeptide) and an excess of nutrients (e.g. glucose, NEFAs) trigger toll-like receptor (TLR) and IL-1 signalling, initiating NF-κB-mediated expression of inflammatory genes such as *IL1B*, *IL18* and *NLRP3* [13–18]. The NLRP3 protein senses intracellular PAMPs and DAMPs, including a wide array of metabolically important signalling molecules (see the following section). To reinstate tissue homeostasis, the inflammasome initiates a defence response in the form of cytokine release with or without inflammatory cell death (pyroptosis) [19]. In a second step, NLRP3 assembles with ASC and pro-caspase-1 to form a functional complex termed an ‘inflammasome’, with pro-caspase-1 proteolytically cleaving itself, pro-IL-1β and pro-IL-18 to yield active IL-1β and IL-18. In contrast to IL-1β, activation of pro-IL-1α does not require proteolytic cleavage by caspase-1 or NLRP3 assembly [20]. More recently, the importance of gasdermin D in inflammasome-mediated cytokine release has been recognised. Although not restricted to the NLRP3 inflammasome, cleavage of gasdermin D by caspase-1 induces the formation of a membrane pore, which is required for the selective release of active IL-1β [20] and execution of pyroptosis [21]. The structural features of gasdermin D imply that the negatively charged gasdermin D pore repels the negatively charged pro-domains, thus selectively allowing mature IL-1β to be released [20]. Figure 1 provides an overview of IL-1β production and secretion.

Molecularly, downstream of the inflammasome, IL-1α and IL-1β bind the IL-1 receptor (IL-1R) 1, competing with IL-1Ra to induce downstream IL-1 signalling in the presence of the accessory protein IL-1R3. Although all nucleated cells express the IL-1R1, not all cell types express IL-1R3, which is essential for signal transduction. IL-1α and IL-1β also bind the decoy receptor IL-1R2 but this fails to induce transmembrane signalling.

Thus, IL-1α and IL-1β signalling are counterbalanced by two endogenous inhibitors (IL-1Ra and IL-1R2), and the ratio of IL-1 agonists to IL-1Ra determines the response downstream of the IL-1R1 in target tissues. Overactivation of the IL-1 pathway leads to uncontrolled IL-1 amplification via IL-1β-induced IL-1β [22], as observed in autoinflammatory diseases. In individuals with type 2 diabetes, this is further amplified by endogenous downregulation of IL-1Ra [23].

## Pathological activation of IL-1β by metabolic stress, ageing and the microbiome

**Fig. 2:**
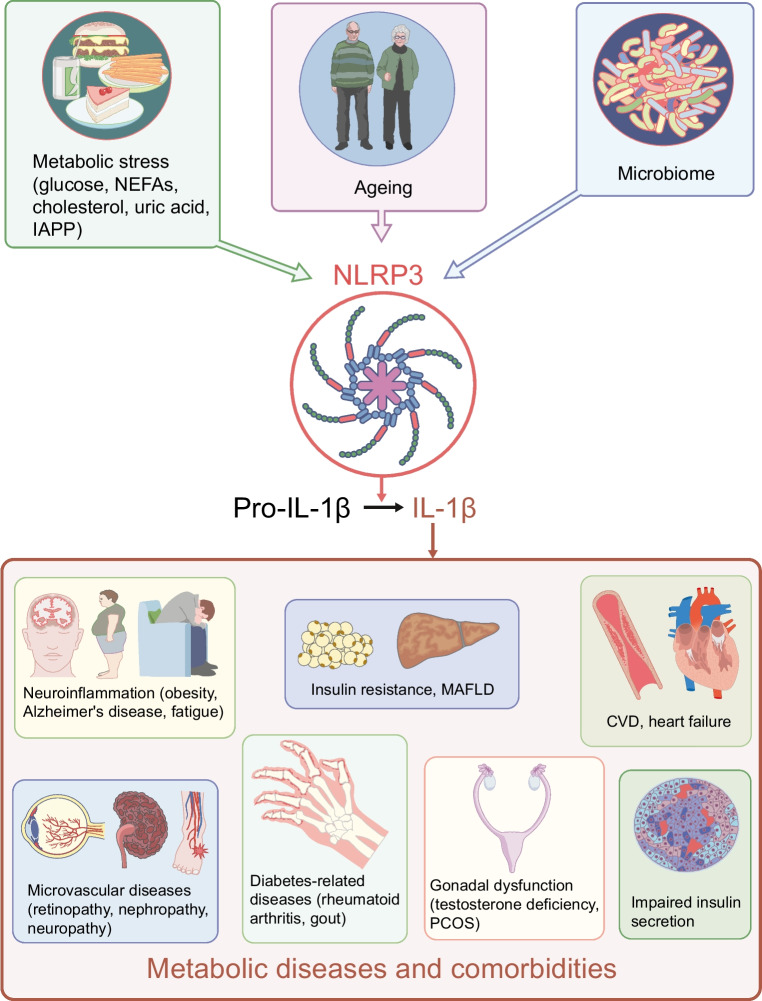
Role of the NLRP3 inflammasome–IL-1β pathway in the pathogenesis of metabolic diseases and comorbidities. Metabolic stress induced by NEFAs, glucose, cholesterol, uric acid and islet amyloid polypeptide (IAPP), along with ageing and changes in the microbiome, activate NLRP3 with subsequent cleavage of pro-IL-1β. In turn, IL-1β contributes to impaired insulin secretion, insulin resistance, MAFLD, atherosclerosis, heart failure, retinopathy, nephropathy, neuropathy, obesity, Alzheimer’s disease, fatigue, testosterone deficiency, polycystic ovary syndrome, rheumatoid arthritis and gout. Blocking IL-1 signalling or inhibition of NLRP3 counteracts pathologies associated with metabolic diseases. This figure is available as part of a downloadable slideset

For decades, the role of cytokines, including IL-1β, in pancreatic islets was studied primarily in relation to pathology and islet dysfunction. The first description of insulitis in type 1 diabetes dates back to 1902, when mononuclear cell infiltration in the islet of a child who died in ketoacidosis was reported [24]. However, the mechanistic link between immune cells and beta cell destruction was discovered much later. Almost 40 years ago, in the context of autoimmune type 1 diabetes, prolonged IL-1β exposure was shown to impair insulin secretion in isolated islets [25] and to induce beta cell toxicity [26, 27]. Later, in the context of type 2 diabetes, elevated glucose levels were shown to induce IL-1β production [28] (Fig. 2). This was initially observed in isolated human pancreatic islets, along with increased expression of IL-1β in islets from individuals with type 2 diabetes [28]. At first, beta cells themselves were thought to be the islet cellular source of glucose-induced IL-1β. Later, increased numbers of islet macrophages were identified in individuals with type 2 diabetes [29], leading to the suggestion that biologically relevant IL-1β is produced by macrophages. With the discovery of the inflammasome [11], it was shown that hyperglycaemia-induced IL-1β in islets is mediated by this complex [28, 30]. This does not rule out the possibility that beta cells may activate IL-1β via NLRP1 in certain circumstances [31].

Glucose alone does not appear to be sufficient to promote IL-1β activation and release. Consistent with the ‘two-step’ concept of inflammasome activation, macrophages sense the availability of increased energy, via glucose or directly via intracellular ATP, and a second signal is then necessary to induce IL-1β activation [32]. This second signal may be associated with obesity and diabetes and includes cholesterol, uric acid, NEFAs and hypoxia [11, 30, 33–37] (Fig. 2). Of note, while overt hyperglycaemia occurs only in the later stages of diabetes development, small glucose spikes are likely to occur in the early stages and may contribute to inflammasome activation. Within the islets, the human beta cell product islet amyloid polypeptide is a potent inducer of IL-1β [38, 39].

Another player in inflammasome activation is the microbiome (Fig. 2). Thereby, increased intestinal leakage plays an important role, allowing bacterial products (LPS) or even whole bacteria to enter the circulation and promote inflammasome activity [40, 41]. The magnitude of this activation may vary according to the composition of the microbiota [42].

Diabetes incidence increases with age and correlates with increasing activity of the innate immune system (‘inflammaging’) [43]. Accordingly, in ageing islets, IL-1Ra expression decreases while IL-1β expression increases, contributing to the age-associated decline in beta cell function [43] (Fig. 2).

## Role of IL-1β in defective insulin secretion

The detection of IL-1β and identification of increased numbers of islet macrophages in human type 2 diabetes and animal models of type 2 diabetes [28, 29] made it conceivable that insulitis plays a role in this condition. Interestingly, in metabolically healthy mice, islet macrophages already exhibit a proinflammatory phenotype, a state that persists throughout the development of obesity and type 2 diabetes [44]. Today, it is well established that metabolic stress (elevated levels of glucose, cholesterol, uric acid and NEFAs) increases the numbers of islet immune cells and cytokine levels (reviewed in detail [45]), triggering chronic low-grade islet inflammation. To restore normoglycaemia in a hyperglycaemic state, beta cells increase insulin secretion. However, this increased workload can eventually lead to beta cell exhaustion, a reversible state induced by chronic high secretory demand associated with inflammasome activation and elevated levels of IL-1β [28, 30], and ultimately to beta cell failure with impaired insulin secretion. Mechanistically, this might be mediated by secretion-induced oxidative and endoplasmic reticulum stress, as well as increased levels of the amyloid precursor islet amyloid polypeptide, which is co-secreted with insulin from beta cells and activates the NLRP3 inflammasome [38, 39].

These proinflammatory processes also initiate the secretion of chemokines from endocrine cells, which, in turn, recruit more immune cells to the islets [46]. Further, given the auto-stimulatory role of IL-1β [22] and the downregulation of islet IL-1Ra via glucotoxicity [47], these processes overall initiate a self-propelling proinflammatory phenotype. Consequently, reducing IL-1 signalling by either macrophage depletion or blocking by IL-1Ra improves insulin secretion [48, 49]. Chronic low-grade inflammation in ageing impairs beta cell proliferation [47]. In addition, IL-1β and IL-1β-dependent cytokines have been shown to promote beta cell dedifferentiation in cultured human and mouse islets [50]. Thus, part of the insulin secretion defects associated with prolonged inflammation might be mediated by impaired beta cell mass and beta cell dedifferentiation. Importantly, tissue remodelling is associated with inflammation and, in states of increased secretory demand, such as in obesity-induced metabolic stress or pregnancy, an initial inflammatory state may be needed for proper adaptation of beta cell mass [51, 52]. Further, macrophages and their secretory products are needed for proper tissue development. Mice that lack macrophages show impaired beta cell mass and function [53]. When islets are damaged, macrophages switch their phenotype and release cytokines and various other factors that support regeneration, such as TGF-β, EGF and IGF-1, inducing proliferation and beta cell survival [54].

## Physiological role of the IL-1β pathway

The primary role of inflammation is to promote adaptation in response to environmental insults and to restore tissue homeostasis. The master cytokine IL-1β plays a crucial role in these processes. Analysis of the expression pattern of the IL-1R paved the way for studies on the physiological effect of IL-1 signalling in immunometabolism. NLRP3 expression in hematopoietic stem cells has been described as a physiological and essential signal for cell expansion, development and release from bone marrow to peripheral blood [55]. IL-1β also has a prominent role in acute physiological processes. As such, IL-1β administration was found to induce insulin secretion and hypoglycaemia [56–58]. In a fasting–refeeding model, myeloid cell-derived IL-1β promoted meal-induced insulin secretion. Moreover, injection of IL-1β per se induced insulin secretion in an islet IL-1R-dependent manner and promoted glucose uptake into immune cells [32]. Further, *Il1r1* knockout mice were unable to increase insulin secretion in response to a high-fat diet [59]. Wiedemann et al identified that neuronally derived IL-1β mediates insulin secretion in anticipation of food intake [60]. This cephalic-phase insulin secretion was shown to be impaired in individuals with obesity as well as in mice fed a high-fat diet [60]. Similarly, islets isolated from healthy donors but not from individuals with type 2 diabetes secrete insulin in response to IL-1β [59]. Glucose-induced IL-1β was also shown to amplify pathological insulin secretion in response to a meal after bariatric surgery. Consequently, hypoglycaemic episodes in these individuals were prevented when glucose levels were lowered by SGLT2 inhibition or IL-1β was blocked by antagonising the IL-1R [61]. These data suggest that acute and transient increases in IL-1β contribute to insulin secretion, but that chronic low-grade inflammation as observed in obesity desensitises beta cells in an IL-1-dependent manner. This is supported by the fact that dampening IL-1β prevents high-fat-diet-induced deterioration of cephalic-phase insulin secretion [60]. It is noteworthy that other IL-1-related cytokines such as IL-33, IL-6 and IL-1Ra also directly or indirectly regulate insulin secretion [47, 62, 63].

## Circulating levels of IL-1β in correlation with onset of diabetes and its complication

IL-1β is produced locally and acts in a paracrine manner. It is one of the most potent cytokines and therefore even very low concentrations can induce IL-1 signalling in target cells [64]. Serum IL-1β is bound to proteins and its concentration is generally too low for robust detection by standard assays. Therefore, circulating IL-1β levels do not necessarily reflect the level of tissue inflammasome activity; instead, they are likely to reflect spillover of local production of IL-1β. Accordingly, even when inflamed, small organs such as pancreatic islets are unlikely to contribute to circulating levels of IL-1β, in contrast to adipose tissue or the liver. Therefore, tissue samples should be obtained to determine the level of IL-1β activity. Measurement of IL-1-dependent inflammatory markers such as C-reactive protein (CRP), IL-6, IL-8 and IL-1Ra is also informative. This can be complemented by analysis of leucocyte counts and, even better, IL-1β expression and release from peripheral blood mononuclear cells [65]. However, clinical studies with specific IL-1 antagonists and including functional outcomes are warranted for definitive conclusions.

With these caveats in mind, interesting epidemiological studies have observed elevated circulating levels of IL-1β and IL-1-dependent CRP, IL-6 and IL-1Ra in individuals with type 2 diabetes [66–68]. Elevated levels of these factors have also been shown to predict the development of type 2 diabetes [66, 68–72] and CVD [66–68, 73]. Importantly, IL-1 antagonism reduces levels of CRP, IL-6 and leucocytosis in those with type 2 diabetes [74].

## IL-1β: friend or foe?

The above descriptions of the physiological and pathological roles of IL-1β in glucose regulation seem to contradict each other. This can be reconciled as follows. The role of IL-1β as an insulin secretagogue during feeding [32] can lead to beta cell exhaustion. This is supported by the observation that K^+^ channel openers reduce insulin secretion, ultimately improving insulin production in individuals with type 2 diabetes by reducing the workload of beta cells [75]. Furthermore, chronic stimulation of beta cells by IL-1β may promote unprocessed proinsulin secretion with subsequent obesity and diabetes [76]. Therefore, the benefit of IL-1 antagonism in those with type 2 diabetes may result from ‘beta cell rest’. In addition, chronic elevation of IL-1β can lead to deleterious inflammatory processes [22]. Furthermore, upregulation of IL-1β leading to elevated insulin levels may become unfavourable for metabolism, as insulin reinforces the proinflammatory state of macrophages [32]. Finally, excessive IL-1β stimulation may lead to resistance to IL-1β signalling, which can be corrected by blocking IL-1 [60]. Indeed, islets from individuals with type 2 diabetes fail to respond to IL-1β with insulin secretion, whereas islets from healthy donors are reactive to IL-1β [59].

## IL-18: a key regulator of body weight?

The role of IL-18 in energy and glucose homeostasis is not yet well understood. Overexpression of IL-18-binding protein or global deletion of IL-18 or the IL-18 receptor led to obesity, hyperglycaemia and insulin resistance in 5- to 7-month-old mice [77–79]. Exogenous IL-18 administration decreased food intake and improved insulin tolerance in mice [77, 78]. The alterations mentioned above were observed in mice fed a chow or low-fat diet, while divergent results were observed in mice fed a high-fat diet. In this scenario, IL-18 and IL-18 receptor knockout mice showed increased body weight gain or no alteration, respectively [78, 79]. These results suggest that IL-18 acts as a homeostatic regulator, opposing the energy surplus, but more data are needed to contextualise these conflicting results. This may have implications for delineating the effects of inflammasome inhibition.

## Clinical translation

### Glucose-lowering effects

The role of IL-1β in insulin regulation described above has been translated into clinical studies. An initial trial in individuals with type 2 diabetes showed that anakinra (recombinant human IL-1Ra) improved beta cell secretory function and reduced HbA_1c_ [74]. Several follow-up studies have confirmed the ability of anakinra to improve impaired insulin secretion [80, 81]. Anakinra has a short half-life and requires daily dosing. In addition, injection site reactions are common (although they resolve after 2–4 weeks of treatment). Therefore, antibodies against IL-1β have been developed that allow for monthly to quarterly dosing. Studies with these antibodies have confirmed the benefit of antagonising IL-1β in type 2 diabetes, with a placebo-corrected reduction in HbA_1c_ of up to 0.9%. (14 mmol/mol). However, the magnitude of the effects varied depending on baseline HbA_1c_ levels and sample sizes [82–85] (Table 1).

**Table 1:** Clinical studies of NLRP3 inhibition or IL-1 antagonism for the treatment of type 2 diabetes and its complications

Drug	Study	Effect	Comment
IL-1R antagonist (anakinra)	Interleukin‑1‑receptor antagonist in type 2 diabetes mellitus [74] Sustained effects of interleukin‑1 receptor antagonist treatment in type 2 diabetes [90]	HbA_1c_ ↓, IL-6 ↓ leucocytes ↓, CRP ↓ Insulin secretion ↑	Follow-up for 39 weeks showed sustained decrease in CRP, increase in insulin secretion and decrease in insulin requirements
IL-1R antagonist (anakinra)	Treatment with anakinra improves disposition index but not insulin sensitivity in nondiabetic subjects with the metabolic syndrome: a randomized, double-blind, placebo-controlled study [80]	Insulin secretion ↑	Individuals without diabetes
Anti-IL-1β antibody (gevokizumab)	Effects of gevokizumab on glycaemia and inflammatory markers in type 2 diabetes [83]	HbA_1c_ ↓, CRP ↓ Insulin secretion ↑	High basal HbA_1c_ Strong effects on HbA_1c_
Anti-IL-1β antibody (canakinumab)	Effect of anti-IL-1beta antibody (canakinumab) on insulin secretion rates in impaired glucose tolerance or type 2 diabetes: results of a randomized, placebo-controlled trial [82]	Insulin secretion ↑ CRP↓	Low basal HbA_1c_
Anti-IL-1β antibody (canakinumab)	Impact of interleukin-1beta antibody (canakinumab) on glycaemic indicators in patients with type 2 diabetes mellitus: results of secondary endpoints from a randomized, placebo-controlled trial [140]	CRP ↓, HbA_1c_ ↓ Insulin secretion ↑(not statistically significant)	Underpowered
Anti-IL-1β antibody (LY2189102)	Double-blind, randomized study evaluating the glycemic and anti-inflammatory effects of subcutaneous LY2189102, a neutralizing IL-1beta antibody, in patients with type 2 diabetes [84]	HbA_1c_ ↓, CRP ↓ Insulin secretion ↑	Further improvement in HbA_1c_ at week 24
IL-1R antagonist (anakinra)	Anakinra treatment in patients with gout and type 2 diabetes [124]	HbA_1c_ ↓	Three cases
IL-1R antagonist (anakinra)	Efficacy of inhibition of IL-1 in patients with rheumatoid arthritis and type 2 diabetes mellitus: two case reports and review of the literature [141]	HbA_1c_ ↓	Two cases
Anti-IL-1β antibody (canakinumab)	Anti-inflammatory therapy with canakinumab for the prevention and management of diabetes [87]	Prevention of diabetes for 4 years HbA_1c_ ↓, CRP ↓	Preventive effect lost during follow-up Improvement in HbA_1c_ lost after change in glucose-lowering drugs Cardiovascular benefit
IL-1R antagonist (anakinra)	Anti-interleukin-1 treatment in patients with rheumatoid arthritis and type 2 diabetes (TRACK): a multicentre, open-label, randomised controlled trial [85]	HbA_1c_ ↓	Strong effects on HbA_1c_, possibly due to concomitant improvement in rheumatoid arthritis
NLRP3 inhibitor (dapansutrile)	Phase 1B, randomized, double-blinded, dose escalation, single-center, repeat dose safety and pharmacodynamics study of the oral NLRP3 inhibitor dapansutrile in subjects with NYHA II-III systolic heart failure [89]	Fasting glucose ↓	Subanalysis

In a large cardiovascular outcomes study (Canakinumab Anti-inflammatory Thrombosis Outcomes Study [CANTOS]), treatment with an anti-IL-1β antibody prevented cardiovascular events [86]. A subanalysis of the 40% of participants with diabetes showed that blocking IL-1β also significantly decreased HbA_1c_ during the first 6–9 months of treatment, with the effect waning over the course of the study [87]. This attenuation of the effect may be due to the design of the study, which allowed for lifestyle interventions and adjustments to standard glucose-lowering therapies after the first visits. In support of this notion, anti-IL-1β antibodies decreased HbA_1c_ throughout the study in those with prediabetes (increased HbA_1c_ or impaired fasting glucose). However, IL-1β inhibition did not reduce the rates of new-onset diabetes. Nevertheless, a detailed analysis of the study revealed that IL-1 antagonism did prevent new-onset diabetes for 4 years. After this period, the number of participants who were further followed in the study decreased by 90% and thus the effect of anti-IL-1β antibody was no longer detectable. The observed prevention of diabetes over 4 years suggests that an inflammatory process is already underway in those with prediabetes.

A meta-analysis of 2921 participants with type 2 diabetes treated with an IL-1-blocking therapy showed a highly significant reduction in HbA_1c_ (*p*<0.00001 [88]).

More recently, a subanalysis of a Phase Ib study using the oral NLRP3 inhibitor dapansutrile showed a statistically significant reduction in fasting blood glucose from 7.7 to 5.9 mmol/l after 12 days of treatment [89].

### Disease modification: islet function

Beyond the glucose-lowering effect of blocking IL-1β, inhibition of the inflammasome–IL-1β pathway may have a disease-modifying effect, that is, prevention of the deterioration of insulin secretion as a result of irreversible beta cell damage. This notion is supported by preclinical studies showing that blocking NLRP3 or IL-1β activity can prevent beta cell death and preserve beta cell function. In humans with type 2 diabetes, 3 months of anakinra treatment increased insulin secretion and decreased the ratio of proinsulin to insulin [74], indicating improved beta cell function. Interestingly, the ratio remained improved for 39 weeks after anakinra withdrawal [90]. However, longer studies are needed to show the durability of these effects and the extent to which this would allow beta cell regeneration.

### Effects on diabetes complications: CVD, MAFLD, retinopathy, nephropathy and neuropathy

As mentioned above, the NLRP3 inflammasome is a critical sensor of metabolic changes. Indeed, the NLRP3 inflammasome is activated by elevated levels of glucose, NEFAs, cholesterol, uric acid and hypoxia [11, 30, 33–37]. Depending on the specific tissue involved, inflammasome activation leads to distinct pathologies and can be observed in CVD, metabolic dysfunction-associated fatty liver disease (MAFLD, formerly known as non-alcoholic fatty liver disease [NAFLD]), retinopathy, nephropathy and neuropathy, among others.

Chronic activation of the innate immune system, as reflected by mildly elevated levels of CRP, IL-6 and circulating leucocytes, is strongly correlated with increased cardiovascular complications. In people with type 2 diabetes, anakinra reduces all three of these factors [74]. The causal relationship between this IL-1β-dependent activation of innate immunity and cardiovascular events was demonstrated in the CANTOS study, which included more than 10,000 participants treated with an anti-IL-1β antibody [86]. Interestingly, although participants were selected on the basis of CRP levels and the presence of CVD, the vast majority had impaired glucose metabolism, supporting the link between inflammation and diabetes. This was substantiated by the observed decrease in HbA_1c_ during the first 6–9 months of treatment described above. This also suggests that some individuals selected on biomarkers reflecting chronic inflammasome activation may particularly benefit from this therapeutic approach.

A subanalysis of the CANTOS trial showed that IL-1β antagonism also reduced hospitalisations for heart failure and mortality risk [91]. The effect was greatest in those with a higher BMI and diabetes. These data confirmed previous proof-of-concept studies by Abbate and colleagues [92–95]. In a follow-up Phase Ib study, improvements in left ventricular ejection fraction and in exercise time were observed in individuals with heart failure treated with the oral NLRP3 inhibitor dapansutrile [89].

Another potentially clinically relevant effect is the ability of IL-1 antagonism to lower blood pressure in individuals with obesity. Indeed, in a placebo-controlled trial, anakinra significantly lowered blood pressure, possibly because of the observed increase in vasodilatory angiotensin peptide (1–7) and decrease in peripheral vascular resistance [96].

Inflammation is critical in the pathogenesis of diabetic retinopathy [97, 98]. An important effector molecule in this pathology is vascular endothelial growth factor, which can be induced directly by IL-1β, but also by hypoxia and other cytokines. Similarly, preclinical studies suggest an important role for NLRP3 activation in diabetic nephropathy [99]. Finally, strong associative clinical data link the IL-1 pathway with diabetic neuropathy [100]. Therefore, clinical studies are warranted to assess the importance of the NLRP3 inflammasome–IL-1β pathway in diabetic retinopathy, chronic kidney disease and polyneuropathy (Fig. 2).

### Brain effects: obesity, Alzheimer’s disease and fatigue

Type 2 diabetes and obesity are associated with inflammasome-mediated activation of inflammatory pathways in the central nervous system [101]. This inflammation may contribute to obesity, Alzheimer’s disease and fatigue.

Indeed, mice deficient in NLRP3 are protected from high-fat-diet-induced obesity [102]. A recent follow-up study tested the efficacy of two NLRP3 inhibitors in reversing obesity in mice [103]. The body weight-lowering effect of the inhibitors was compared with caloric restriction and the GLP-1 receptor agonist semaglutide, and NLRP3 inhibition reduced obesity to a similar extent. Importantly, NLRP3 inhibition and GLP-1 agonism appear to be synergistic, pointing to either different modes of action or restoration of GLP-1 receptor sensitivity, which could be impaired by chronic inflammation. Interestingly, two other NLRP3 inhibitors, VTX3232 and dapansutrile, appear to have similar effects on body weight, although the data are not yet published [104]. These impressive preclinical effects on body weight of four different NLRP3 inhibitors were not observed in human studies using IL-1 antagonists [74]. However, this may be because of the lack of brain penetration of IL-1Ra and anti-IL-1β antibodies, unlike inhibitors of NLRP3. An alternative explanation is the body weight effect of IL-18, which is altered by inhibitors of NLRP3 but not by IL-1 antagonism [105–108]. Planned and ongoing clinical studies with dapansutrile (NCT06047262) and VTX3232 will show whether these drugs decrease body weight in humans.

Alzheimer’s disease is strongly associated with diabetes and chronic neuroinflammation [109]. Mechanistically, this inflammation may be partly due to metabolic stress and amplified by the aggregation of amyloid fibrils [110], in a striking similarity to the islet inflammation described above. Activation of IL-1β may play an important role in this process [111]. Accordingly, pharmacological inhibition of NLRP3 in a mouse model of Alzheimer’s disease rescued cognitive impairment [112].

Fatigue has a major impact on quality of life and is mediated in part by IL-1β [113]. Individuals with type 2 diabetes are prone to fatigue, which can be improved by inhibiting IL-1β [114]. In support of the role of IL-1β in metabolic stress-induced fatigue, IL-1 antagonism reduces postprandial fatigue, particularly in individuals with obesity [115] (Fig. 2).

### Gonadal dysfunction: testosterone deficiency and polycystic ovary syndrome

Obesity and type 2 diabetes also contribute to gonadal dysfunction, at least in part because of increased inflammasome activation. In men, IL-1β inhibits testosterone secretion by reducing gonadotropin production and by directly inhibiting testicular androgen production [116]. Accordingly, treatment with anakinra in obese men with testosterone deficiency increases testosterone levels [117].

The ovary expresses the IL-1R with cyclical variation, consistent with the notion that it has effects on ovulation [118]. In addition, women with polycystic ovary syndrome show overactivity of the IL-1 system [119]. In a pilot study in individuals with polycystic ovary syndrome, anakinra reduced hyperandrogenaemia and favoured the onset of ovulatory cycles [120] (Fig. 2).

### Effects on diabetes-related diseases: rheumatoid arthritis and gout

Type 2 diabetes is associated with other inflammatory diseases in which the IL-1 system is involved, such as gout and rheumatoid arthritis. This makes it possible to treat more than one disease with one therapeutic agent. This was demonstrated in a multicentre study of individuals with rheumatoid arthritis and diabetes [85]. Treatment with anakinra for 6 months improved both HbA_1c_ and rheumatic disease activity. In these individuals, anakinra was particularly effective (HbA_1c_ decreased by >1%), probably because the NLRP3 inflammasome is activated by both diseases.

Uric acid is a strong inducer of the NLRP3 inflammasome [33]. Consequently, IL-1β mediates gouty arthritis [121] and IL-1β antagonism is highly effective in treating this condition [122, 123]. Some people are affected by both gout and type 2 diabetes; in this situation, blocking the IL-1 system appears to be highly efficient at lowering fasting blood glucose and HbA_1c_ and improving gout, as reported in case reports and anecdotally [124].

Taking advantage of the fact that IL-1 antagonism is approved in several countries for the treatment of gout and rheumatoid arthritis, clinicians may already use this anti-inflammatory strategy in the presence of concomitant type 2 diabetes. However, dedicated studies with more attractive agents such as oral NLRP3 inhibitors are warranted before this strategy can be widely recommended (Fig. 2).

### Safety

The safety of IL-1 antagonists is well established. This may be due to normalisation of persistent inflammasome activity as observed in type 2 diabetes [125]. Thus, more than 100,000 people with rheumatoid arthritis have been treated with anakinra and infections have rarely been reported. Similarly, anti-IL-1β antibodies can be considered safe except in cases of severe infection that require intensive care, when they are associated with an increased mortality risk [86]. This has not been observed with anakinra, probably because of its short half-life, which allows for immediate withdrawal in case of an infection.

NLRP3 inhibitors also appear to be safe and have a short half-life, allowing for rapid discontinuation of treatment in the event of side effects. However, these small molecules act intracellularly and are therefore more likely to have off-target effects than IL-1 antagonists. The processing of pro-IL-1β to IL-1β is mainly NLRP3-dependent [126]. However, alternative pathways are able to release active IL-1β via the inflammasomes NLRP1, NLRC4 (NLR family CARD domain-containing protein 4) and AIM2 (absent in melanoma 2) [126], caspase-8 and other enzymes [127–132], with reduced efficiency. Thus, in contrast to pharmacological blockage of IL-1β or the IL-1 receptor, administration of NLRP3 inhibitors is not expected to completely block IL-1β activity, regardless of the potency of the drug. Further, one might argue that blocking NLRP3 could cause disturbances because of decreased IL-18 levels, although no human disease has been linked to IL-18 deficiency [133]. Notably, elevated IL-18 levels observed in individuals with obesity [17, 105, 107, 134, 135] are associated with IL-18 resistance [135] and decrease following weight loss [105–107]. Additionally, it has been demonstrated that NLRP1-derived IL-18 prevents obesity [108], suggesting that sources other than the NLRP3 inflammasome contribute substantially to IL-18 production. Thus, although there is no scientific reason to expect severe side effects with pharmacological NLRP3 inhibition, long-term safety studies with NLRP3 inhibitors still need to be performed.

## Perspective

Obesity and diabetes are associated with a large number of diseases. Thereby, overnutrition-induced activation of the inflammasome appears to play an important role. Blocking the subsequent IL-1β activation provides an opportunity to improve the health of affected individuals. Those most likely to benefit from such a strategy can be identified by the ‘fingerprint’ of IL-1 activity, which includes elevated serum concentrations of CRP, IL-6, IL-8 and IL-1Ra, elevated leucocyte counts and a proinflammatory expression pattern in peripheral blood mononuclear cells.

The potential of this therapeutic approach has been clinically demonstrated for IL-1 antagonism. It is already being used clinically in some cases of type 2 diabetes and concomitant gout or rheumatoid arthritis, for which anakinra and anti-IL-1β antibodies are approved.

However, mainly because of patent expirations, it is unlikely that pharmaceutical companies will seek approval for IL-1 antagonism to treat diabetes and its complications. Nonetheless, there are several NLRP3 inhibitors in clinical development that may find their way into the clinic. Several companies have developed small orally active molecules that can target NLRP3 [136–138]. Some molecules are suspected not to inhibit the NLRP3 inflammasome [139], but this seems to depend on the evaluation method used [136].

The clinically most advanced NLRP3 inhibitor is dapansutrile, which is currently being tested in a 6 month trial of diabetes and related complications (ClinicalTrials.gov NCT06047262).

## Conclusion

In conclusion, inhibition of the NLRP3 inflammasome–IL-1β pathway in obesity and type 2 diabetes may represent an attractive way to target the pathogenesis of these diseases and their complications. Dedicated clinical studies are underway and are expected to provide answers to the questions outlined in this review in the near future.

## Supplementary Information

Below is the link to the electronic supplementary material.

Slideset of figures (PPTX 532 KB)

## Abbreviations

ASC: Apoptosis-associated speck-like protein containing a caspase recruitment domain
CANTOS: Canakinumab Anti-inflammatory Thrombosis Outcomes Study
CRP: C-reactive protein
IL-1R: IL-1 receptor
IL-1Ra: IL-1 receptor antagonist
LPS: Lipopolysaccharide
MAFLD: Metabolic dysfunction-associated fatty liver disease
NLRP3: NACHT, LRR and PYD domains-containing protein 3
TLR: Toll-like receptor
