# Supplementary material for: Targeting the NLRP3 inflammasome–IL-1β pathway in type 2 diabetes and obesity
Source: Diabetologia. 2024 Nov 4;68(1):3–16. doi: 10.1007/s00125-024-06306-1 (PMC11663173; doi:10.1007/s00125-024-06306-1)
Supplement: Supplementary file 1 — Slideset of figures (PPTX 532 KB) [file 125_2024_6306_MOESM1_ESM.pptx]

## Slide 1
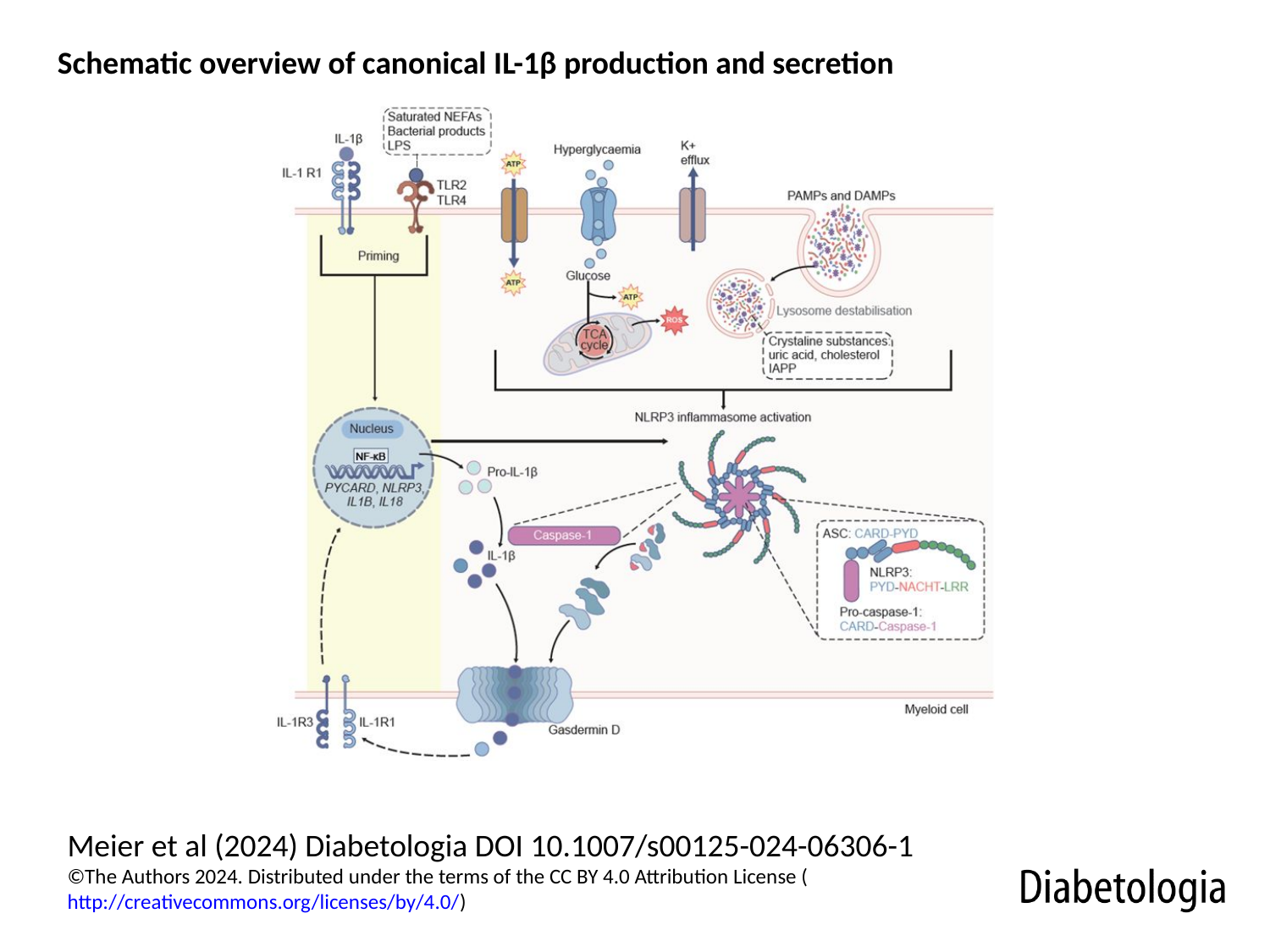

Schematic overview of canonical IL-1β production and secretion
Meier et al (2024) Diabetologia DOI 10.1007/s00125-024-06306-1
©The Authors 2024. Distributed under the terms of the CC BY 4.0 Attribution License (http://creativecommons.org/licenses/by/4.0/)

## Slide 2
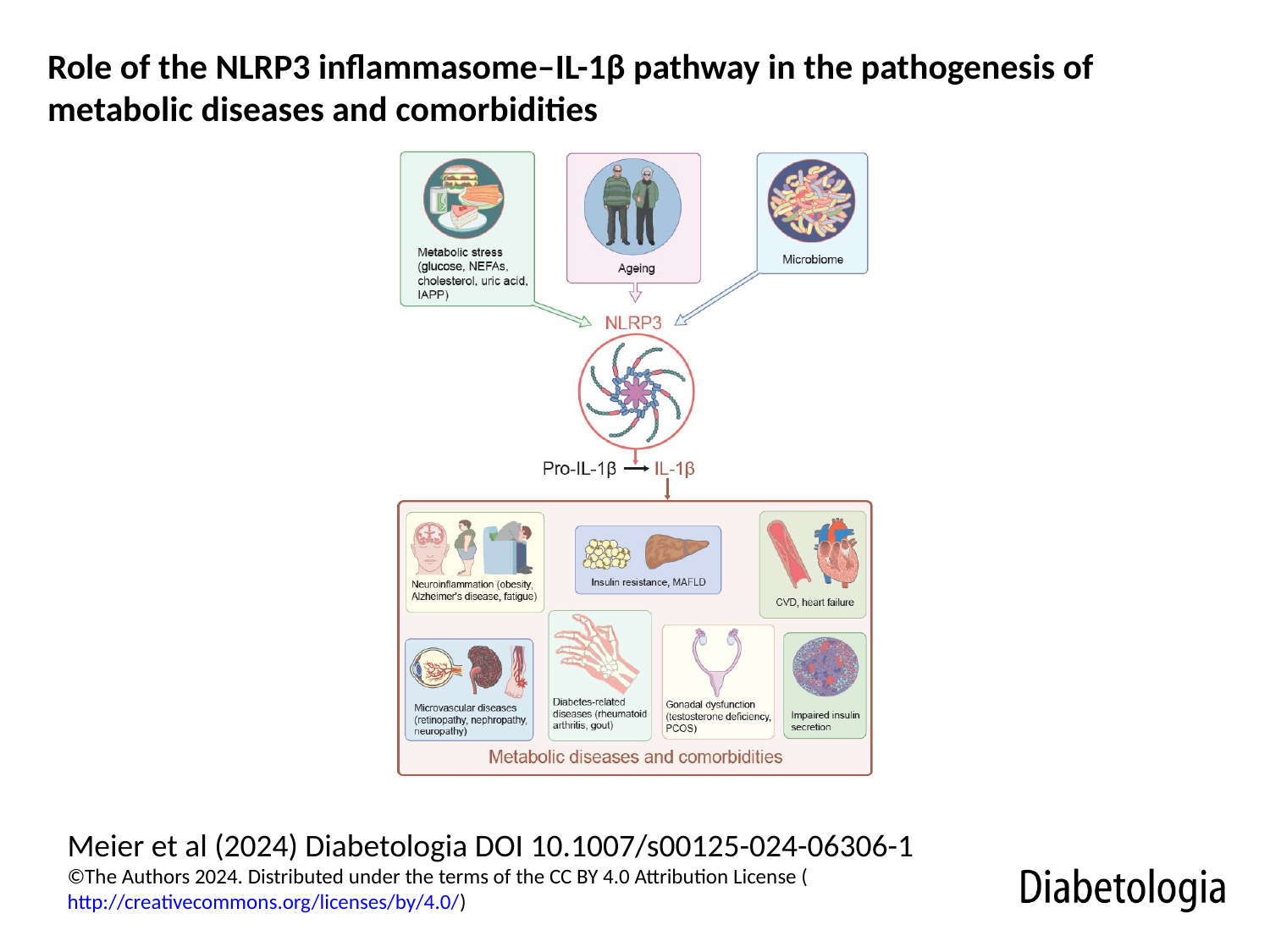

Role of the NLRP3 inflammasome–IL-1β pathway in the pathogenesis of metabolic diseases and comorbidities
Meier et al (2024) Diabetologia DOI 10.1007/s00125-024-06306-1
©The Authors 2024. Distributed under the terms of the CC BY 4.0 Attribution License (http://creativecommons.org/licenses/by/4.0/)
